# Association between Hyperosmolar Hyperglycemic State and Venous Thromboembolism in Diabetes Patients: A Nationwide Analysis in Taiwan

**DOI:** 10.3390/jpm12020302

**Published:** 2022-02-17

**Authors:** Wei-Ting Wei, Shu-Man Lin, Jin-Yi Hsu, Ying-Ying Wu, Ching-Hui Loh, Huei-Kai Huang, Peter Pin-Sung Liu

**Affiliations:** 1 Department of Emergency Medicine, Hualien Tzu Chi Hospital, Buddhist Tzu Chi Medical Foundation, Hualien 970, Taiwan; timduncan1001@gmail.com; 2 Department of Physical Medicine and Rehabilitation, Hualien Tzu Chi Hospital, Buddhist Tzu Chi Medical Foundation, Hualien 970, Taiwan; drshumanlin@gmail.com; 3 School of Medicine, Tzu Chi University, Hualien 970, Taiwan; hsu.jinyi@gmail.com (J.-Y.H.); twdoc1960@gmail.com (C.-H.L.); 4 Center for Aging and Health, Hualien Tzu Chi Hospital, Buddhist Tzu Chi Medical Foundation, Hualien 970, Taiwan; 5 Department of Psychiatry, Hualien Tzu Chi Hospital, Buddhist Tzu Chi Medical Foundation, Hualien 970, Taiwan; squirrellyying@gmail.com; 6 Department of Medical Research, Hualien Tzu Chi Hospital, Buddhist Tzu Chi Medical Foundation, Hualien 970, Taiwan; 7 Department of Family Medicine, Hualien Tzu Chi Hospital, Buddhist Tzu Chi Medical Foundation, Hualien 970, Taiwan; 8 Institute of Medical Sciences, Tzu Chi University, Hualien 970, Taiwan

**Keywords:** hyperosmolar hyperglycemic state, venous thromboembolism, deep vein thrombosis, pulmonary embolism

## Abstract

Background: Previous studies in Western countries have shown that a hyperosmolar hyperglycemic state (HHS) is associated with an increased risk of venous thromboembolism (VTE); in these cases, prophylactic anticoagulant treatment is suggested. However, the association between HHS and VTE in Asian populations remains undetermined. Therefore, we aimed to evaluate whether HHS is associated with an increased risk of VTE in diabetic Taiwanese patients. Methods: This nationwide, population-based, retrospective cohort study was conducted using the Taiwan National Health Insurance Research Database. We enrolled a total of 4,723,607 admission records of patients with diabetes diagnosed with one or more of seven common diseases (pneumonia, urinary tract infection, sepsis, heart disease, stroke, malignancy, and respiratory tract disease) between 2001 and 2018 in Taiwan. The patients were divided into two groups based on the presence (*n* = 46,000) or absence (*n* = 4,677,607) of HHS. We estimated the adjusted odds ratio (aOR) for developing VTE within 90 days after the index hospitalization using multivariable logistic regression with generalized estimating equations accounting for repeated measures. Results: Overall, patients admitted with HHS had a similar risk of VTE compared with those admitted without HHS (408/46,000 vs. 39,345/4,677,607; aOR = 1.06, 95% CI: 0.97–1.17, *p* = 0.190). A similar non-significant association between HHS and VTE was found regardless of age and sex subgroups. Conclusions: There was no significant association between HHS and overall VTE risk in patients with diabetes in Taiwan. The results of our study may not support the use of prophylactic anticoagulant therapy in diabetic Taiwanese patients with HHS.

## 1. Introduction

Venous thromboembolism (VTE) is a major global health concern. This condition consists of two types: deep vein thrombosis (DVT) and pulmonary embolism (PE). The annual incidence of VTE in Asia varies from 14 to 20 per 100,000 individuals [1]. Hospitalized patients have a higher risk of VTE than non-hospitalized patients [2]. VTE can be a dangerous disease that, without early diagnosis and prompt intervention, results in a fatality rate of up to 30% [3,4,5].

Hyperosmolar hyperglycemic state (HHS) is a complication of diabetes mellitus (DM), characterized by severe hyperglycemia, hyperosmolality, and dehydration without significant ketoacidosis. The mortality rate of HHS can be as high as 20% [6,7,8]. The precipitating factors in the development of HHS include infection, discontinuation or inadequate insulin therapy, pancreatitis, myocardial infarction, cerebrovascular accident, and some specific drug use [6].

Several case reports have described VTE development during the HHS [9,10,11,12]. Severe dehydration and hyperosmolarity, resulting in the release of tissue thromboplastins from disrupted endothelial cells, may enhance coagulation [13]. In the American population, patients hospitalized for HHS were shown to have a higher risk of VTE than those with uncomplicated DM [14]. The current guidelines for the management of HHS recommend prophylactic anticoagulation for the full duration of hospitalization in all patients with HHS, unless contraindications exist [15]. However, the benefits and risks of pharmacological prophylaxis for VTE in patients with HHS have not been proven in clinical trials, Ref. [16,17] and current evidence is insufficient to support this recommendation in the Asian population. Since the risk of VTE in Asians is significantly lower than that in Westerners, [1] whether HHS increases the risk of VTE and should be treated with prophylactic anticoagulants in Asian populations is still undetermined. To date, patients with HHS or most medically ill patients do not routinely receive prophylactic anticoagulants during hospitalization in Taiwan. Therefore, this study aimed to evaluate whether HHS is associated with an increased risk of VTE among hospitalized patients with DM in Taiwan.

## 2. Materials and Methods

### 2.1. Data Sources

This nationwide retrospective cohort study was conducted using data from the National Health Insurance Database (NHID). The National Health Insurance (NHI) program was started in Taiwan in 1995 and enrolled >99% of the Taiwanese population, approximately 23 million people, and 97% of the hospitals and clinics [18]. The NHI of Taiwan contains abundant health and medical treatment information on insured individuals, such as inpatient and outpatient care services, emergency department services, dental care, use of traditional Chinese medicine, and other medical care [19,20,21]. The diagnostic codes used in the NHID conformed to the International Classification of Diseases, 9th Revision, Clinical Modification (ICD-9-CM) codes before 2016, and ICD-10-CM codes after 2016. The data of each participant was anonymized by encrypting the identification number. Patient characteristics and medical records were obtained from the Health and Welfare Data Science Center of Taiwan’s Ministry of Health and Welfare. This study was conducted in accordance with the World Medical Association Declaration of Helsinki: Ethical Principles for Medical Research Involving Human Subjects. This study was approved by the Research Ethics Committee of Hualien Tzu Chi Hospital (REC No: IRB107-152-C), and the requirement for informed consent was waived.

### 2.2. Study Population

We initially identified all admissions with DM, defined as those with prescriptions of antidiabetic drugs (Anatomical Therapeutic Chemical [ATC] code: A10) during admission or within 90 days prior to admission, between 2001 and 2018 in Taiwan. To ensure the comparability between study groups, we limited the admissions to those with the main diagnoses of the following common conditions: pneumonia, urinary tract infection (UTI), sepsis, heart disease, stroke, malignancy, and respiratory tract disease (other than pneumonia) (Supplemental Table S1), which were found to be common diagnoses and associated with the precipitating factor of HHS among hospitalized DM patients in previous studies [9,17]. We divided our study population into admissions with and without the diagnosis of HHS (ICD-9: 250.2; ICD-10: E08.0, E11.0, E13.0). Patients who had been diagnosed with VTE or prescribed anticoagulants (ATC codes: B01AA, B01AB, B01AE, B01AF) within 90 days prior to admission were excluded. Patients who died within 90 days after admission without VTE diagnosis were excluded.

### 2.3. Outcome Measures

The primary outcome was defined as the occurrence of VTE within 90 days after the index date; the index date was defined as the date of admission with DM as aforementioned. The VTE included both DVT (ICD-9-CM codes: 453.2, 453.4, 453.5, 453.7, 453.8, and 453.9; ICD-10-CM codes: I82.2, I82.4, I82.5, I82.60, I82.62, I82.89, and I82.9) and PE (ICD-9-CM codes: 415.1 except for 415.11 and 415.12; ICD-10-CM codes: I26 except for I26.01 and I26.90). The accuracy of the ICD-9-CM codes for diagnosing VTE has been previously validated in Taiwan, with high positive predictive values [22]. We also evaluated the occurrence of DVT and PE as two separate secondary outcomes. In addition to the overall analysis, the comparison of VTE risk between HHS and non-HHS admissions was performed based on the subgroups of the main admission diagnoses (pneumonia, UTI, sepsis, heart disease, stroke, malignancy, and respiratory tract disease) [23].

### 2.4. Covariates

The baseline and clinical characteristics included age, sex, comorbidities, medications, and income. A pre-existing comorbidity, according to the ICD-9-CM and ICD-10-CM codes, was defined as a disease that was diagnosed during at least one hospital admission or two outpatient visits in the year prior to the index date. The Charlson Comorbidity Index was calculated to represent the overall comorbidity status [24]. Data on monthly income was retrieved to serve as socioeconomic status and was categorized into four levels (≥40,000, 20,000–39,999, 1–19,999 New Taiwan dollars, or financially dependent) based on income-related NHI premiums.

### 2.5. Statistical Analysis

We used the standardized mean difference to evaluate the differences in baseline characteristics between study groups. Since each admission event was considered as a unit of analysis, some of the recorded admissions may have involved the same patient and should be considered as repeated-measure data. Therefore, we estimated the odds ratio (OR) for developing VTE using logistic regression models with generalized estimating equations accounting for the potential correlation between repeated measures within subjects. Multivariable analyses were performed with adjustment for all covariates listed in Table 1, including age, sex, income level, major diagnoses, Charlson Comorbidity Index, comorbidities, and medication use. A two-sided probability value of <0.05 was considered statistically significant. Data management and statistical analyses were performed using Stata, version 14 (Stata Corporation LLC; College Station, TX, USA) and SAS, version 9.4 (SAS Institute, Inc.; Cary, NC, USA).

## 3. Results

### 3.1. General Characteristics

Overall, 4,723,607 admission records were enrolled in this study; of these, 46,000 and 4,677,607 belonged to the HHS and non-HHS admission groups, respectively. The mean age was 70.6 years in the HHS group and 68.7 years in the non-HHS group. The top three main admission diagnoses were heart disease, sepsis, and respiratory tract disease in the HHS group and heart disease, malignancy, and respiratory tract disease in the non-HHS group. Some baseline characteristics were different between study groups, such as a higher proportion of stroke but lower proportion of coronary artery disease, hyperlipidemia, and malignancy in the HHS group than in the non-HHS group. The detailed baseline characteristics are shown in Table 1.

### 3.2. Association between HHS and VTE

Overall, the event rates of VTE were 408/46,000 (8.87‰) and 39,345/4,677,607 (8.41‰) for the HHS and non-HHS groups, respectively. There was no significant association between HHS admission and the development of VTE in both univariable and multivariable analyses (crude OR = 1.06, 95% CI: 0.97–1.17, *p* = 0.196; adjusted OR [aOR] = 1.06, 95% CI: 0.97–1.17, *p* = 0.190) (Table 2).

In the age- and sex-stratified analyses, a similar non-significant association between HHS and VTE was found, regardless of age (<65 years and ≥65 years) and sex subgroups (Table 3). The tests for interaction also supported that no subgroup effect of age/sex existed on the association between HHS and VTE.

Regarding the secondary outcomes, patients admitted with HHS were slightly more likely to develop DVT than those admitted without HHS (event rate: 6.00‰ vs. 5.56‰; aOR = 1.16, 95% CI: 1.03–1.31, *p* = 0.012). On the other hand, there was no significant association between HHS and PE (event rate: 3.35‰ vs. 3.09‰; aOR = 0.97, 95% CI: 0.83–1.12, *p* = 0.641) (Table 4).

### 3.3. Analyses According to the Main Admission Diagnoses

The association between HHS and the development of VTE was not significant in the subgroups of patients admitted due to pneumonia, sepsis, heart disease, stroke, malignancy, and respiratory tract disease (Table 5). However, we found a significant association between HHS and VTE in patients admitted for UTI (aOR = 1.70, 95% CI: 1.19–2.51, *p* = 0.008).

Regarding the secondary outcomes, HHS was significantly associated with DVT development only in patients admitted for UTI (aOR = 1.95, 95% CI: 1.26–3.03, *p* = 0.003), but not in those admitted for the other main diagnoses (Supplementary Table S2). No significant association between HHS and PE was found in the analyses for all subgroups of the main admission diagnoses (Supplementary Table S3).

## 4. Discussion

This nationwide large-scale cohort study evaluated the association between HHS and development of VTE within 90 days after admission. We found that patients with diabetes hospitalized with HHS were not at a higher risk of VTE than those without HHS. This non-significant association between HHS and VTE was consistent in different age and sex subgroups. To our knowledge, this is the first Asian study using nationwide data to evaluate the association between HHS and VTE development in patients with DM.

To date, there has been limited evidence regarding the association between HHS and the risk of VTE. A previous retrospective cohort study retrieved from the California Patient Discharge Data Set showed that diabetic patients with hyperosmolarity were at an increased risk of developing VTE during their inpatient stay [14]. Moreover, the risk of VTE in HHS was higher than that in uncomplicated DM (hazard ratio [HR] = 3.0; 95% CI: 2.1–4.5) [14]. A typical patient presenting with HHS often presents with other afflictions, such as infection, heart disease, and cerebrovascular accident; however, the aforementioned study did not explore this characteristic, which may have interfered with the results. Overall, our study results, based on the entire Taiwanese population, are inconsistent with those of the American study. Our study addressed the knowledge gap regarding the association between HHS and VTE in an Asian population.

Epidemiology data in Taiwan suggest that DM is a risk factor for developing VTE [25,26]. DM is regarded as a hypercoagulable state [27,28]. The exact mechanism of VTE among patients with DM is complicated and includes platelet and endothelial cell dysfunction, [29,30] coagulative activation, [31,32] and suppression of fibrinolysis [33]. Patients with HHS had more severe dehydration, which is theoretically prone to a hypercoagulable state; however, in our study, the HHS group did not have an increased risk of overall VTE compared with the non-HHS group. The results may be explained by the aggressive fluid replacement that occurs during treatment to rapidly correct the hyperosmolarity and restore intravascular volume, which corrects dehydration and may avoid a hypercoagulable state. Moreover, Asians may have more efficient fibrinolytic activity and lower hereditary thrombophilia, leading to a reduced hypercoagulable state [34,35,36]. Further studies are necessary to verify the exact underlying mechanisms to re-examine the proposed hypotheses. Although a statistically significant association was found between HHS and DVT risk in our secondary outcome analysis, the risk was only slightly increased (event rate: 6.00‰ vs. 5.56‰). This difference was not sufficient to support the use of prophylactic anticoagulant treatment in patients with HHS. Of note, the subgroup analyses incidentally showed that a significantly higher risk of VTE was found specifically in patients with HHS who were admitted with a UTI, but not in those with other main diagnoses (pneumonia, sepsis, heart disease, stroke, malignancy, or respiratory tract disease). The exact underlying mechanism of this finding is still unknown, and further studies are still needed to illustrate the association between HHS and VTE in patients admitted for UTI.

Although further evidence is still needed to strengthen our findings, they hold significant implications. Previous studies have revealed that Asians have a significantly lower risk of developing VTE than Western populations [1,37,38]. Therefore, the association between HHS and VTE derived from Western populations is unlikely to be directly applicable to Asian groups. Prophylactic anticoagulation was suggested for HHS hospitalization in Western countries, where the benefit and bleeding risk may not be the same as those found in Asia. Although some conditions (such as malignancy, pneumonia, and heart failure) are considered risk factors for VTE, prophylactic anticoagulants are not routinely prescribed in Taiwan [39,40,41,42]. Our study further supported that, unlike in Western populations, prophylactic anticoagulants seem unnecessary for patients with HHS in Taiwan. Although a slightly elevated DVT risk may be observed in patients with HHS, clinicians should weigh the pros and cons (e.g., risk of major bleeding) of anticoagulant treatment. Our study findings may not support the routine use of prophylactic anticoagulant therapy for patients with HHS in Taiwan [43].

The main advantage of the present study was the large sample size included in this nationwide population-based design. However, this study has some limitations. First, some clinical data, such as patient lifestyle, cognition level, physical function, body mass index, and laboratory test results, could not be collected; thus, unmeasured factors may have confounded the results. Second, personal identifiers were encrypted by the Health and Welfare Data Science Center in Taiwan. Indeed, we only obtained anonymous data and were unable to confirm diagnostic accuracy by visiting patients directly, but instead relied on claims-based data. However, the diagnostic accuracy of VTE, the main study outcome, was proven to have high positive predictive values in Taiwan [22]. Third, although the diagnoses and treatment of DM, as well as other comorbidities, may have changed over time, our analyses did not consider the potential differences over varying periods. Further studies are required to address this issue. Fourth, this study was conducted in Taiwan and its generalizability to other Asian countries is still questionable.

In conclusion, this nationwide large-scale cohort study demonstrated that there was no significant association between HHS and overall VTE risk among patients with DM in Taiwan. Although a slightly higher risk of DVT was found in patients with HHS, the magnitude of the increase was small. Our study may, thus, not support the use of prophylactic anticoagulant therapy in Taiwanese diabetic patients with HHS. Further research is warranted to confirm this finding for the development of evidence-based guidelines.

## Figures and Tables

**Table 1:** General characteristics of admission records with and without HHS.

Variables	HHS		Non-HHS		SMD
	(*n* = 46,000)		(*n* = 4,677,607)		
	*n*	%	*n*	%	
Age, mean ± SD	70.6 ± 13.7		68.7 ± 12.9		0.1481
Sex					
Male	23,356	50.8	2,509,758	53.7	0.0577
Female	22,644	49.2	2,167,849	46.4	0.0577
Income level (NTD)					
Dependent	14,553	31.6	1,370,942	29.3	0.0506
15,840–24,999	21,802	47.4	2,116,458	45.3	0.0431
25,000–44,999	6160	13.4	773,223	16.5	0.0881
≥ 45,000	3485	7.6	416,984	8.9	0.0484
Major admission diagnosis					
Pneumonia	7525	16.4	507,713	10.9	0.1612
Urinary tract infection	2893	6.3	198,460	4.2	0.0919
Sepsis	9835	21.4	387,022	8.3	0.3754
Heart disease	10,548	22.9	1,301,622	27.8	0.1128
Stroke	3349	7.3	443,937	9.5	0.0798
Malignancy	2485	5.4	1,019,051	21.8	0.4925
Respiratory tract disease	9365	20.4	819,802	17.5	0.0723
Charlson Comorbidity Index, mean ± SD	3.1 ± 2.4		3.8 ± 2.8		0.2603
Comorbidities					
Hypertension	29,633	64.4	3,231,659	69.1	0.0993
Atrial fibrillation	2128	4.6	246,327	5.3	0.0295
Stroke	14,113	30.7	1,117,407	23.9	0.1529
Heart failure	5917	12.9	688,511	14.7	0.0540
Coronary artery disease	10,264	22.3	1,377,629	29.5	0.1636
COPD	7907	17.2	886,982	19.0	0.0460
Chronic renal failure	5810	12.6	677,667	14.5	0.0543
Cirrhosis	1942	4.2	267,805	5.7	0.0695
Hyperlipidemia	10,953	23.8	1,426,584	30.5	0.1508
Fracture of lower limbs	1927	4.2	151,134	3.2	0.0508
Gout	3173	6.9	381,512	8.2	0.0478
Malignancy	4877	10.6	1,104,759	23.6	0.3510
Thyroid dysfunction	675	1.5	79,384	1.7	0.0184
Baseline medication use					
Statins	10,799	23.5	1,408,819	30.1	0.1503
Antiplatelet	20,027	43.5	2,202,503	47.1	0.0714

Continuous data are expressed as mean ± SD, and categorical data are expressed as numbers and percentages. Abbreviations: COPD = chronic obstructive pulmonary disease; HHS = hyperosmolar hyperglycemic state; NTD = New Taiwan Dollar; SD = standard deviation; SMD = standardized mean difference.

**Table 2:** Event rate and odds ratio of VTE in HHS versus non-HHS admissions.

	HHS	Non-HHS
Admission numbers	46,000	4,677,607
VTE events	408	39,345
Event rate (‰)	8.87	8.41
Univariate model		
Crude OR (95% CI)	1.06 (0.97–1.17)	1 (ref.)
*p* value	0.196	
Multivariable model *		
Adjusted OR (95% CI)	1.06 (0.97–1.17)	1 (ref.)
*p* value	0.190	

* Multivariable logistic regression model adjusting for all baseline characteristics shown in Table 1. Abbreviations: HHS = hyperosmolar hyperglycemic state; OR = odds ratio; VTE = venous thromboembolism; CI = confidence interval; ref. = reference.

**Table 3:** Odds ratio of VTE in HHS versus non-HHS admissions stratified by age and sex.

	Univariate Model		Multivariable Model *		
	Crude OR (95% CI)	*p* Value	Adjusted OR (95% CI)	*p* Value	*p* for Interaction
Age < 65 years					0.540
HHSNon-HHS	1.02 (0.84–1.24)	0.832	1.04 (0.86–1.26)	0.676	
	1.00 (ref)		1.00 (ref)		
Age ≥ 65 years					
HHS	1.08 (0.98–1.20)	0.138	1.11 (1.00–1.23)	0.058	
Non-HHS	1.00 (ref)		1.00 (ref)		
Male					0.670
HHS	1.03 (0.90–1.19)	0.659	1.05 (0.91–1.21)	0.484	
Non-HHS	1.00 (ref)		1.00 (ref)		
Female					
HHS	1.08 (0.96–1.22)	0.219	1.10 (0.98–1.25)	0.116	
Non-HHS	1.00 (ref)		1.00 (ref)		

* The multivariable logistic regression model adjusting for all baseline characteristics shown in Table 1. Abbreviations: HHS = hyperosmolar hyperglycemic state; OR = odds ratio; VTE = venous thromboembolism; CI = confidence interval; ref. = reference.

**Table 4:** Event rate and odds ratio of DVT and PE in HHS versus non-HHS admissions.

	Event Rate (‰)		Univariate Model		Multivariable Model *	
			Crude OR (95% CI)	*p* Value	Adjusted OR (95% CI)	*p* Value
DVT						
HHS	6.00		1.09 (0.97–1.23)	0.137	1.16 (1.03–1.31)	0.012
Non-HHS	5.56		1.00 (ref.)		1.00 (ref.)	
PE						
HHS	3.35		1.07 (0.93–1.23)	0.353	0.97 (0.83–1.12)	0.641
Non-HHS	3.09		1.00 (ref.)		1.00 (ref.)	

* The multivariable logistic regression model adjusting for all baseline characteristics shown in Table 1. Abbreviations: HHS = hyperosmolar hyperglycemic state; OR = odds ratio; DVT = deep vein thrombosis; PE = pulmonary embolism; OR = odds ratio; CI = confidence interval; ref. = reference.

**Table 5:** Odds ratio of VTE in HHS versus non-HHS admissions based on different admission diagnoses.

Main Diagnosis	Group	Univariate Model		Multivariable Model *	
		Crude OR (95% CI)	*p* Value	Adjusted OR (95% CI)	*p* Value
Pneumonia	HHS	1.13 (0.89–1.44)	0.305	1.17 (0.91–1.49)	0.220
	Non-HHS	1.00 (ref.)		1.00 (ref.)	
Urinary tract infection	HHS	1.77 (1.19–2.63)	0.005	1.70 (1.15–2.51)	0.008
	Non-HHS	1.00 (ref.)		1.00 (ref.)	
Sepsis	HHS	1.07 (0.89–1.30)	0.465	1.09 (0.89–1.33)	0.396
	Non-HHS	1.00 (ref.)		1.00 (ref.)	
Heart disease	HHS	0.95 (0.76–1.18)	0.625	0.91 (0.74–1.13)	0.415
	Non-HHS	1.00 (ref.)		1.00 (ref.)	
Stroke	HHS	1.49 (0.99–2.24)	0.058	1.43 (0.95–2.16)	0.086
	Non-HHS	1.00 (ref.)		1.00 (ref.)	
Malignancy	HHS	1.22 (0.92–1.62)	0.175	1.19 (0.89–1.58)	0.237
	Non-HHS	1.00 (ref.)		1.00 (ref.)	
Respiratory tract disease	HHS	0.87 (0.70–1.09)	0.236	0.88 (0.70–1.10)	0.264
	Non-HHS	1.00 (ref.)		1.00 (ref.)	

* The multivariable logistic regression model adjusting for all baseline characteristics shown in Table 1. Abbreviations: HHS = hyperosmolar hyperglycemic state; OR = odds ratio; VTE = venous thromboembolism; CI = confidence interval; ref. = reference.

## Data Availability

The dataset used in this study is managed by the Taiwan Ministry of Health and Welfare and thus cannot be made available publicly. Researchers interested in accessing this dataset can submit a formal application to the Ministry of Health and Welfare to request access (Taiwan Ministry of Health and Welfare, No. 488, Section 6, Zhongxiao E Rd, Nangang District, Taipei 115, Taiwan; website: https://dep.mohw.gov.tw/DOS/cp-2516-59203-113.html. The last accessing date: 2 February 2022).

## Supplementary Materials

The following are available online at https://www.mdpi.com/article/10.3390/jpm12020302/s1, Table S1: Main admission diagnosis codes, Table S2: Odds ratio of DVT in HHS versus non-HHS admissions based on different main admission diagnoses, Table S3: Odds ratio of PE in HHS versus non-HHS admissions based on different main admission diagnoses.
