# Supplementary material for: Association between Hyperosmolar Hyperglycemic State and Venous Thromboembolism in Diabetes Patients: A Nationwide Analysis in Taiwan
Source: J Pers Med. 2022 Feb 17;12(2):302. doi: 10.3390/jpm12020302 (PMC8880712; doi:10.3390/jpm12020302)
Supplement: Supplementary file 1 [file jpm-12-00302-s001.zip › jpm-1581714-supplementary.pdf]

Journal of Personalized Medicine

MDPI

Supplementary Materials

Table S1. Main admission diagnosis codes.

Diagnosis	ICD-9-CM Codes	ICD-10-CM Codes
Sepsis	790.7, 038.xx, 995.91, 995.92, 785.52	R78.81, A40, A41.9, R65.20, A41, R65.21
Respiratory tract disease	460.xx-466.xx, 470.xx-478.xx, 490.xx-496.xx, 487.xx-488.xx, 500.xx-508.xx, and 510.xx-519.xx	J00-J11, J19-J99
Heart disease	398.91, 402.xx, 404.xx, 410.xx-414.xx, 420.xx-428.xx	I09.81, I11, I13, I21-I25, I32-I50
Pneumonia	480.xx-486.xx	J12-J18
Stroke	430.xx-436.xx	I60-I67
Malignancy	140.xx-208.xx	C00-C96, Z51
Urinary tract infection	590.1, 590.2, 559.0	N10, N15.1, N39.0

Abbreviations: ICD-9-CM = International Classification of Diseases, 9th Revision, Clinical Modification; ICD-10-CM = International Classification of Diseases, 10th Revision, Clinical Modification.

Table S2. Odds ratio of DVT in HHS versus non-HHS admissions based on different main admission diagnoses.

Main Diagnosis	Group	Univariate Model		Multivariable Model †	
		Crude OR (95% CI)	*p* Value	Adjusted OR (95% CI)	*p* Value
Pneumonia	HHS	1.06 (0.75–1.49)	0.739	1.11 (0.79–1.57)	0.534
	Non-HHS	1.00 (ref.)		1.00 (ref.)	
Urinary tract infection	HHS	2.02 (1.29–3.17)	0.002	1.95 (1.26–3.03)	0.003
	Non-HHS	1.00 (ref.)		1.00 (ref.)	
Sepsis	HHS	1.16 (0.92–1.47)	0.210	1.24 (0.97–1.57)	0.080
	Non-HHS	1.00 (ref.)		1.00 (ref.)	
Heart disease	HHS	0.99 (0.76–1.27)	0.911	0.98 (0.77–1.26)	0.889
	Non-HHS	1.00 (ref.)		1.00 (ref.)	
Stroke	HHS	1.44 (0.87–2.36)	0.154	1.38 (0.84–2.27)	0.199
	Non-HHS	1.00 (ref.)		1.00 (ref.)	
Malignancy	HHS	1.38 (1.01–1.88)	0.043	1.34 (0.89–1.83)	0.065
	Non-HHS	1.00 (ref.)		1.00 (ref.)	
Respiratory tract disease	HHS	1.02 (0.75–1.40)	0.883	1.05 (0.77–1.44)	0.748
	Non-HHS	1.00 (ref.)		1.00 (ref.)	

† Multivariable logistic regression model with adjustments for all baseline characteristics shown in Table1. Abbreviations: HHS = hyperosmolar hyperglycemic state; OR = odds ratio; DVT = deep venous thrombosis; CI = confidence interval; ref. = reference.

Table S3. Odds ratio of PE in HHS versus non-HHS admissions based on different main admission diagnoses.

Main Diagnosis	Group	Univariate Model		Multivariable Model †	
		Crude OR (95% CI)	*p* Value	Adjusted OR (95% CI)	*p* Value
Pneumonia	HHS	1.25 (0.90–1.73)	0.191	1.24 (0.89–1.74)	0.200
	Non-HHS	1.00 (ref.)		1.00 (ref.)	
Urinary tract infection	HHS	1.43 (0.69–2.96)	0.338	1.36 (0.65–2.83)	0.412
	Non-HHS	1.00 (ref.)		1.00 (ref.)	
Sepsis	HHS	1.02 (0.75–1.40)	0.892	0.94 (0.68–1.30)	0.725
	Non-HHS	1.00 (ref.)		1.00 (ref.)	
Heart disease	HHS	0.96 (0.67–1.37)	0.820	0.87 (0.60–1.26)	0.458
	Non-HHS	1.00 (ref.)		1.00 (ref.)	
Stroke	HHS	1.44 (0.70–2.95)	0.317	1.39 (0.67–2.87)	0.380
	Non-HHS	1.00 (ref.)		1.00 (ref.)	
Malignancy	HHS	1.44 (0.40–1.44)	0.392	0.74 (0.38–1.44)	0.378
	Non-HHS	1.00 (ref.)		1.00 (ref.)	
Respiratory tract disease	HHS	0.78 (0.58–1.07)	0.125	0.77 (0.56–1.06)	0.104
	Non-HHS	1.00 (ref.)		1.00 (ref.)	

† Multivariable logistic regression model with adjustments for all baseline characteristics shown in Table1. Abbreviations: HHS = hyperosmolar hyperglycemic state; OR = odds ratio; PE = pulmonary embolism; CI = confidence interval; ref. = reference.
